# Seasonal variation in Internet searches for vitamin D

**DOI:** 10.1007/s11657-017-0322-7

**Published:** 2017-03-10

**Authors:** Rebecca J. Moon, Elizabeth M. Curtis, Justin H. Davies, Cyrus Cooper, Nicholas C. Harvey

**Affiliations:** 1 MRC Lifecourse Epidemiology Unit, University of Southampton, Southampton, UK; 2 Paediatric Endocrinology, University Hospital Southampton NHS Foundation Trust, Southampton, UK; 3 NIHR Southampton Nutrition Biomedical Research Centre, University of Southampton and University Hospital Southampton NHS Foundation Trust, Southampton, UK; 4 National Institute for Health Research (NIHR) Musculoskeletal Biomedical Research Unit, University of Oxford, Oxford, UK

**Keywords:** Vitamin D, Google Trends, Internet, Epidemiology

## Abstract

***Summary*:**

Internet search rates for “vitamin D” were explored using Google Trends. Search rates increased from 2004 until 2010 and thereafter displayed a seasonal pattern peaking in late winter. This knowledge could help guide the timing of public health interventions aimed at managing vitamin D deficiency.

**Purpose:**

The Internet is an important source of health information. Analysis of Internet search activity rates can provide information on disease epidemiology, health related behaviors and public interest. We explored Internet search rates for vitamin D to determine whether this reflects the increasing scientific interest in this topic.

**Methods:**

Google Trends is a publically available tool that provides data on Internet searches using Google. Search activity for the term “vitamin D” from 1st January 2004 until 31st October 2016 was obtained. Comparison was made to other bone and nutrition related terms.

**Results:**

Worldwide, searches for “vitamin D” increased from 2004 until 2010 and thereafter a statistically significant (*p* < 0.001) seasonal pattern with a peak in February and nadir in August was observed. This seasonal pattern was evident for searches originating from both the USA (peak in February) and Australia (peak in August); *p* < 0.001 for both. Searches for the terms “osteoporosis”, “rickets”, “back pain” or “folic acid” did not display the increase observed for vitamin D or evidence of seasonal variation.

**Conclusion:**

Public interest in vitamin D, as assessed by Internet search activity, did increase from 2004 to 2010, likely reflecting the growing scientific interest, but now displays a seasonal pattern with peak interest during late winter. This information could be used to guide public health approaches to managing vitamin D deficiency.

## Background

In recent years there has been increasing scientific interest in 25-hydroxyvitamin D [25(OH)D] [1] and its potential role in a wide variety of health outcomes. A large number of observational studies have reported associations between 25(OH)D and malignancy, cardiovascular disease, mental illness, diabetes, obstetric outcomes and all-cause mortality, yet there is a lack of high quality evidence from intervention studies to support a causal role for vitamin D in outcomes apart from osteomalacia and rickets [2–4]. Nonetheless, both the testing for biochemical 25(OH)D deficiency [5] and prescribing of vitamin D supplementation [6] have increased.

Many national guidelines now provide advice on daily reference intakes (DRIs) for vitamin D and/or suggest vitamin D supplementation for those with risk factors for biochemically low levels of 25(OH)D [7–9]. Despite this, studies have suggested that the general public have only limited knowledge regarding vitamin D [6, 10–12]; in the UK, the National Institute for Health and Care Excellence (NICE) have highlighted the importance of increasing public knowledge regarding vitamin D [8]. The Internet is now an important source of health information for the general public, and many individuals will frequently use the Internet as first line when in need of health-related information [13]. As such, analysis of Internet search activity has been shown to provide useful information on the epidemiology of disease [14] and changes in health-related behavior [15], and could potentially guide future public health messages. We therefore undertook this study to explore whether the public interest in vitamin D assessed using Internet search activity reflected the increasing scientific interest in this topic.

## Methods

Data for Internet search activity were obtained using Google Trends (www.google.com/trends). This is a publically searchable database which reports the search activity for a term within Google from January 2004 until the present day. Approximately 65% of all Internet searches are performed using Google [16]. Absolute numbers of searches per month are not available, but instead the data are presented relative to the month for which search activity was highest on a 0 to 100 scale (100 being the highest month). Search activity can be assessed for worldwide usage, or specific countries of origin. A report detailing the most frequent countries from which the term of interest is searched is also provided. We obtained Google search activity for the search term “vitamin D” for both worldwide and individual countries of origin from 1st January 2004 until 31st October 2016. Where necessary “vitamin D” was translated into the local language for the country of search. The monthly data were downloaded from Google Trends in .csv format to Microsoft Excel on 11th November 2016. Comparison was made to other bone and nutrition-related search terms. SPSS v24 was used to perform cosinor analysis to assess for seasonal variation in search activity. A *p* < 0.05 was considered statistically significant.

## Results

**Fig. 1:**
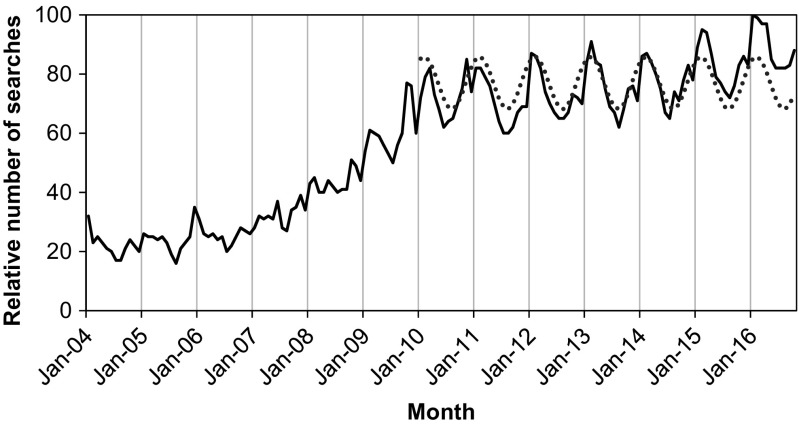
Google search activity for the term “vitamin D”. The *dotted line* represents the seasonal model generated from these data for January 2010 to October 2016

**Fig. 2:**
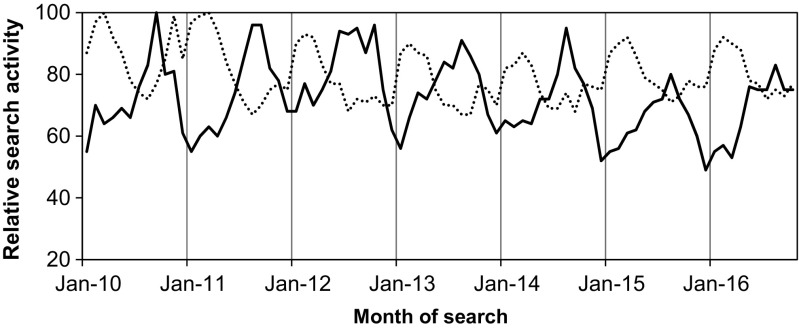
Google search activity for “vitamin D” for searches originating from the USA (*solid line*) and Australia (*dotted line*)

**Fig. 3:**
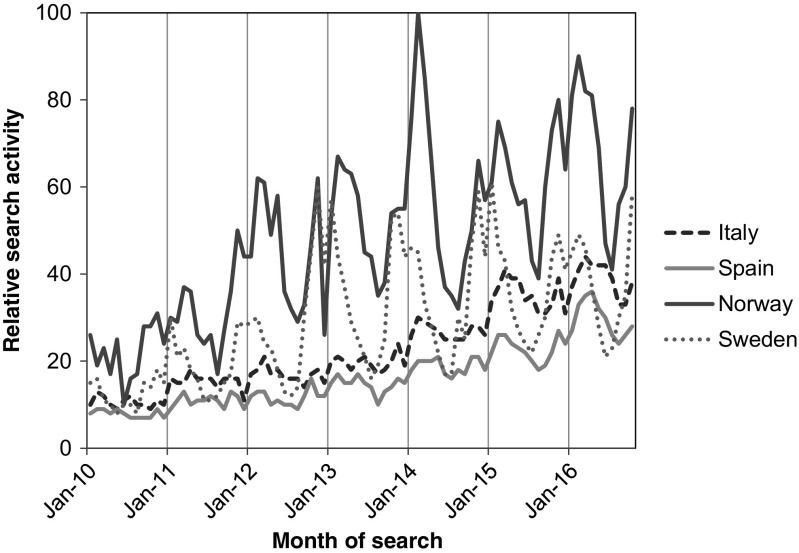
Google search activity for “vitamin D” in Norway and Sweden compared with searches for “vitamina D” in Spain and Italy. All search counts are relative to the month in which the highest number of searches was made in Norway

Worldwide, Google searches for the term “vitamin D” was highest in February 2016. Figure 1 displays the relative number of searches for “vitamin D” per month compared to the number in February 2016. Internet search activity for “vitamin D” increased from 2004 until 2010. Since 2010, there has been a statistically significant (*p* < 0.001) seasonal pattern to Internet searches for “vitamin D” with a peak in February and nadir in August (Fig. 1). The United States of America (USA) and Australia had the highest number of Internet searches for vitamin D for the northern and southern hemispheres, respectively. A seasonal pattern in search activity was evident for both the USA and Australia (*p* < 0.001 for both) but this peaked in February in the USA and August in Australia (Fig. 2). Within Europe, differences in search activity were observed; Sweden and Norway displayed seasonal variation in search activity (*p* < 0.05 for both), whereas searches for “vitamina D” in Spain and Italy occurred less frequently and showed no evidence of seasonality (Fig. 3).

In order to determine if this reflected a seasonal pattern in Internet use, we assessed a number of other musculoskeletal and nutrition-related terms. Google searches for the terms “osteoporosis”, “rickets”, “back pain” or “folic acid” did not display the increase observed with vitamin D, or evidence of seasonal activity for worldwide searches or from the USA or Australia (*p* < 0.05 for all). Conversely, worldwide searches for “calcium” appeared to have two peaks each year in both October and February. A similar pattern was evident for searches originating from the USA, whereas those from Australia did display seasonal variation peaking in June.

## Discussion

The use of the Internet to access information on vitamin D appeared to increase dramatically between 2004 and 2010. Since that time, search activity for vitamin D has varied seasonally, peaking in late winter in both the USA and Australia. Although overall search activity using Google has increased exponentially since 2000 [16], a similar trend was not observed for other musculoskeletal and nutrition related terms we explored, suggesting that this rise in general public interest is likely to reflect the increasing scientific interest, publication rate and media coverage.

It is interesting that Internet searches for vitamin D peak in late winter. We believe that this genuinely reflects an increase in public interest in vitamin D during this time rather than simply reflecting increased Internet usage during colder darker winter months; firstly, searches for “osteoporosis”, “rickets”, “back pain” and “folic acid” did not display seasonal variation and the annual peaks and troughs in searches for “calcium” differed to those for “vitamin D”, and secondly, previous studies have, in contrast, demonstrated seasonal variation in searches for restless leg syndrome and foot and ankle pain peaking in the summer months [17, 18]. Furthermore, searches arising from Norway and Sweden did peak in winter, whereas Spain and Italy had far fewer searches for “vitamina D” recorded and no evidence of seasonal variation in this, perhaps suggesting that at more northerly latitudes interest in vitamin D and/or vitamin D deficiency is greater. One possible explanation for this winter peak could be that individuals with some knowledge on the seasonal variation in vitamin D status are seeking further information to attribute vitamin D to symptoms experienced in winter months, as many people will now obtain health information from the internet prior to attending a medical practitioner [13]. Alternatively, information seeking behavior could be secondary to a health professional suggesting 25(OH)D testing or vitamin D supplementation. These have both increased in recent years [5, 6], but to our knowledge, seasonal variation in requests for 25(OH)D assessment or prescriptions for vitamin D has not been investigated. Nonetheless, this observation of seasonal variation in Internet search activity may have important implications for public health approaches to improving vitamin D status. In particular, if knowledge seeking behaviors with regard to vitamin D are not occurring until late winter, then the opportunity to prevent biochemically low 25(OH)D during the winter months is missed. Jha et al. showed that Google search activity for Fosamax peaked following significant coverage of the drug in the popular media [19], highlighting that such an approach could be used to promote interest and knowledge seeking behaviors for a specific topic. As such, timing vitamin D related health messages for early autumn might encourage supplementation usage and prevent the winter nadir in 25(OH)D, thus suggesting the need to work together with the popular media industry to ensure that information is promoted at the most useful time of the year.

The interpretation of these data is not without limitations. Demographic information on those conducting the searches is not available, and individuals who use the Internet for information seeking may not be representative of the general population or those at risk of vitamin D deficiency. In particular, although Internet usage is increasing amongst older individuals, it remains markedly lower than in younger adults [20]. Nonetheless, this is a population that has actively sought to inform themselves and is therefore likely to be receptive to public health education. However, we do not know which websites were accessed from the Google search, and importantly what messages they promote. Furthermore, it is only possible to speculate on the reason for the searches being undertaken. Finally, this approach can only assess internet searches performed using Google and no other search engines; in particular the use of other search engines may vary between countries, but worldwide Google does account for approximately 65% of all Internet searches [16].

In conclusion, the public interest in vitamin D, as assessed by Internet search activity, initially appeared to mirror the increase in scientific interest, but now displays a seasonal pattern with peak interest during late winter. This information could be used to guide public health approaches to managing vitamin D deficiency.
